# Emerging Role of Podocalyxin in the Progression of Mature B-Cell Non-Hodgkin Lymphoma

**DOI:** 10.3390/cancers12020396

**Published:** 2020-02-08

**Authors:** Estíbaliz Tamayo-Orbegozo, Laura Amo, Javier Díez-García, Elena Amutio, Marta Riñón, Marta Alonso, Paula Arana, Natalia Maruri, Susana Larrucea

**Affiliations:** 1 Regulation of the Immune System Group, Biocruces Bizkaia Health Research Institute, Plaza de Cruces 12, 48903 Barakaldo, Bizkaia, Spain; estitamayo@hotmail.com (E.T.-O.); laura_tpo@hotmail.com (L.A.); 2 Microscopy Facility, Biocruces Bizkaia Health Research Institute, Plaza de Cruces 12, 48903 Barakaldo, Bizkaia, Spain; JAVIER.DIEZGARCIA2@osakidetza.eus; 3 Blood Cancer Group, Biocruces Bizkaia Health Research Institute, Hospital Universitario Cruces, Plaza de Cruces 12, 48903 Barakaldo, Bizkaia, Spain; MARIAELENA.AMUTIODIEZ@osakidetza.eus; 4 Regulation of the Immune System Group, Biocruces Bizkaia Health Research Institute, Hospital Universitario Cruces, Plaza de Cruces 12, 48903 Barakaldo, Bizkaia, Spain; MARTAMARIA.RINONMARTINEZ-GALLO@osakidetza.eus (M.R.); MARTA.ALONSOVARELA@osakidetza.eus (M.A.); p.arana.berganza@gmail.com (P.A.); NATALIA.MARURIMACHADO@osakidetza.eus (N.M.)

**Keywords:** podocalyxin, B-cell non-Hodgkin lymphoma, cancer progression, metastasis, drug resistance, metabolic reprogramming

## Abstract

Mature B-cell non-Hodgkin lymphoma (B-NHL) constitutes a group of heterogeneous malignant lymphoproliferative diseases ranging from indolent to highly aggressive forms. Although the survival after chemo-immunotherapy treatment of mature B-NHL has increased over the last years, many patients relapse or remain refractory due to drug resistance, presenting an unfavorable prognosis. Hence, there is an urgent need to identify new prognostic markers and therapeutic targets. Podocalyxin (PODXL), a sialomucin overexpressed in a variety of tumor cell types and associated with their aggressiveness, has been implicated in multiple aspects of cancer progression, although its participation in hematological malignancies remains unexplored. New evidence points to a role for PODXL in mature B-NHL cell proliferation, survival, migration, drug resistance, and metabolic reprogramming, as well as enhanced levels of PODXL in mature B-NHL. Here, we review the current knowledge on the contribution of PODXL to tumorigenesis, highlighting and discussing its role in mature B-NHL progression.

## 1. Introduction

Non-Hodgkin lymphoma (NHL) represents the most common hematological malignancy in adults worldwide and accounts for approximately 90% of all diagnosed lymphomas in western countries, with B cell-NHL (B-NHL) being more frequent (85%–90%) than T-cell or natural killer (NK)-cell NHL [1,2]. The incidence of NHL has experienced a constant increase in recent years and this disease represents the fifth to ninth most common cancer in the majority of countries worldwide [3]. B-NHL consists of a heterogeneous group of lymphoproliferative malignancies that arise from lymphoid tissue B cells at varying stages of maturation and can spread to other organs, encompassing more than 40 neoplasm subtypes both molecular and clinically different [4]. They are currently classified according to the 2016 revision of the World Health Organization (WHO) classification of lymphoid neoplasms based on cell linage and pathological, genetic, immunophenotypic, and clinical features [4]. Among mature B-NHL, diffuse large B-lymphoma, an aggressive type of NHL, and indolent follicular lymphoma comprise 65% of all NHL and predominate in adulthood [2,5]. Nevertheless, in children, the most common B-NHL is the highly aggressive B-cell neoplasm Burkitt lymphoma [6]. Other mature B-NHLs include chronic lymphocytic leukemia/small lymphocytic lymphoma, mantle cell lymphoma, marginal zone B-cell lymphoma, hairy cell leukemia, and lymphoplasmacytic lymphoma/Waldenström macroglobulinemia [4].

Although the survival in patients with B-NHL has improved significantly during the past two decades since the introduction of anti-CD20 monoclonal antibodies to the standard chemotherapy regimens, many patients are refractory or relapse after treatment due to the acquisition of drug resistance that limits its effectiveness [7,8]. A better understanding of the molecular mechanism governing B-NHL development and chemo-immunotherapy resistance would allow the design of more efficient therapeutic drugs.

PODXL, also known as podocalyxin-like protein 1, PCLP1, PCLP, PC, GCTM-2 antigen, Gp135, or GP200, is a transmembrane protein belonging to the CD34 family of sialomucins and is expressed in multiple normal cell types including podocytes, vascular endothelium, platelets, hematopoietic progenitors, embryonic stem cells, and a subset of neurons [9,10,11,12,13,14,15]. PODXL is connected to the actin cytoskeleton through interaction with ezrin and Na^+^/H^+^-exchanger regulatory factor (NHERF) [16]. Depending on the cellular context, PODXL functions as an anti-adhesive or a pro-adhesive molecule [17,18,19]. PODXL was originally reported as the major apical sialoglycoprotein on kidney glomerular podocytes, where it exerts an anti-adhesive effect that maintains the filtration slits opened by charge repulsion as a result of its highly negative charge [9,17,20,21]. On the contrary, PODXL acts as a pro-adhesive molecule in high endothelial venules, interacting with L-selectin expressed on lymphocytes, suggesting a role for PODXL in lymphocyte recruitment to secondary lymphoid organs [18].

A growing number of studies have implicated PODXL in the development and progression of cancer. Elevated expression of PODXL has been associated with a more aggressive phenotype and poor patient clinical outcome in a variety of human solid cancers [22,23,24,25,26]. Thus far, limited studies have addressed the expression and function of PODXL in hematological neoplasms. PODXL has been found upregulated in mature B-NHL [27] and in blasts from patients with acute lymphoblastic and myeloid leukemia [28,29]. In Burkitt lymphoma cells, PODXL has recently emerged as a molecule that promotes cell proliferation, survival, migration, resistance to chemo-immunotherapy, and metabolism reprogramming [27]. The objective of this review is to summarize the current knowledge on the role of PODXL in tumorigenesis, discussing its contribution to the development of mature B-NHL.

## 2. PODXL Expression and Regulation in Human Malignancies

Expression of PODXL has been detected in numerous human solid malignancies including testicular cancer [30,31,32], breast cancer [22], pancreatic ductal adenocarcinoma [33,34,35], malignant astrocytic tumors [36], lung carcinoma [37,38], undifferentiated thyroid carcinoma [39], renal cell carcinoma [26], colorectal cancer [23,40,41,42], ovarian carcinoma [25], uterine endometrioid adenocarcinoma [43], urothelial bladder cancer [44], glioblastoma multiforme [45], oral squamous cell carcinoma [46,47], hepatocellular carcinoma [48], gastric cancer [49,50,51,52], esophageal carcinoma [50], and prostate cancer [53], as well as in hematological malignancies [27,28,29]. Furthermore, increased PODXL expression has been associated with high-grade tumors and poor clinical outcome in breast cancer [22], colorectal cancer [23,40,42,54], pancreatic ductal adenocarcinoma [35], ovarian cancer [25], renal cell carcinoma [26], urothelial bladder cancer [44,55], glioblastoma multiforme [45], gastric cancer [49,50,51,52], uterine endometrioid adenocarcinoma [43], periampullary adenocarcinoma [56], and esophageal adenocarcinoma [50].

Regarding hematological cancers, the detection of PODXL in normal human hematopoietic cells [57], as well as of Wilms´ tumor antigen 1 (WT1), a transcriptional activator of *PODXL*, in blast cells of the majority of acute myeloid leukemia and acute lymphoblastic leukemia patients [58], prompted the determination of PODXL in leukemia [28,29]. An immunochemical analysis performed in blasts from 81 patients with leukemia showed elevated levels of PODXL expression in 77% of cases of acute myeloid leukemia, 81% of cases of acute lymphoblastic leukemia, and 87% of cases of cutaneous myeloid sarcoma [28], which is a rare extramedullary tumor most often associated with acute myeloid leukemia [59]. Another report revealed by flow cytometry moderate levels of PODXL expression in 15 cases and high levels in 13 cases from a cohort of 73 patients with acute myeloid leukemia, and associated PODXL expression in leukemic blasts with a monocytic immunophenotype, a subtype characterized by poor clinical outcome and short survival [29]. Moreover, the cases of acute myeloid leukemia expressing higher levels of PODXL also displayed increased blast cell counts and higher levels of markers associated with unfavorable prognosis [29]. High levels of PODXL expression were also detected by immunohistochemistry in most of the bone marrow samples obtained from acute myeloid lymphoma patients compared to samples from normal subjects [60].

More recently, we have determined PODXL cell surface expression in malignant cells from a small cohort of patients with B-NHL and in B-NHL cell lines [27]. We found three- to eight-fold higher levels of PODXL expression in malignant cells compared to their normal B-cell counterparts in two out of five cases of follicular lymphoma and one out of three cases of chronic lymphocytic leukemia/small lymphocytic lymphoma. However, no expression of PODXL was observed in two cases of hairy cell leukemia and one case of Waldenström macroglobulinemia [27]. Furthermore, elevated levels of PODXL expression were detected on Raji Burkitt lymphoma cell line and moderate levels on Karpas 422 diffuse large B-cell lymphoma cell line. By contrast, Ramos and Daudi Burkitt lymphoma, Pfeiffer diffuse large B-cell lymphoma, and Karpas 1718 splenic marginal zone lymphoma cell lines showed no expression of PODXL [27].

Human PODXL protein is encoded by *PODXL*, a gene located on chromosome 7q32.3 [61]. A study identified a locus on chromosome 7q32-q33 associated with aggressive forms of prostate cancer using linkage analysis and allelic imbalance techniques [62]. Furthermore, a variable in-frame deletion and a missense variant of *PODXL* were associated with increased risk of prostate and tumor aggressiveness [24]. Interestingly, gain of 7q32.3-q33 region has been shown to predict the risk of disease transformation in patients with aggressive forms of follicular lymphoma [63]. Similarly, a comparative genomic hybridization study involving 46 patients diagnosed with Burkitt lymphoma detected gains on 7q31-q36 or 7q32-q36 regions in three patients and identified the association of gains on 7q with an adverse prognosis [64]. Hence, the increased PODXL levels detected in malignant cells of B-NHL patients and in B-cell lines from our study might be caused by copy number gains of *PODXL* gene or gain mutations.

PODXL expression is positively regulated by WT1 [65] and specific protein 1 (SP1) [66]. WT1, a potent transcriptional regulator of several genes involved in growth, cellular metabolism, and renal differentiation, is highly expressed in many cancers, including hematological malignancies [67]. SP1 plays an important role in several physiological processes such as cell cycle, growth control, apoptosis, angiogenesis, and tumor cell metabolism [68].

PODXL expression can be repressed by some regulatory factors, including tumor suppressor p53 [69], particularly interesting new cysteine-rich protein 1 (PINCH1) [70], and Kruppel-like factor 4 (KLF4) [51]. PINCH1 is an adaptor protein that controls integrin-mediated cell adhesion, migration and epithelial–mesenchymal transition (EMT) and that acts as a transcriptional suppressor of PODXL in podocytes by interacting and inhibiting WT1-induced PODXL expression [70]. KLF4, a member of the KLF family of zinc finger transcription factors that regulates cell proliferation, differentiation, and survival, represses PODXL expression in human gastric cancer cells by directly binding to the 5´UTR of *PODXL* [51]. 

Additionally, epigenetic processes such as DNA methylation and the synthesis of specific microRNAs contribute to the modulation of PODXL expression. The in vitro CpG methylation of *PODXL* promoter resulted in a drastic reduction of its activity in human embryonic kidney (HEK293) cells [66]. In oral squamous cell carcinoma cell lines, hypomethylation of *PODXL* promoter has been associated with aggressiveness [46]. MicroRNAs are small noncoding RNAs that control gene expression post-transcriptionally, and their levels are frequently altered in many tumors, acting both as oncogenes and tumor suppressors. A study showed that miR199b, a microRNA targeting PODXL and DDR1 (discoidin domain receptor 1), regulates the expression of PODXL in K562 chronic myeloid leukemia cell line overexpressing miR-199b and established an inverse correlation between miR199b levels and PODXL expression in patients with acute myeloid leukemia [60]. In another report, the analysis of molecular and clinical data of 166 patients with acute myeloid leukemia from The Cancer Genome Atlas revealed a correlation between low expression of PODXL-targeting miR-199b and poor survival outcome [71]. Regarding B-cell lymphomas, various epigenetic mechanisms have been implicated in the development of these malignancies, including dysregulation of DNA methylation and histone modifications, as well as aberrant expression of microRNAs [72]. Among the most common microRNAs, miR-155, miR-17-92 cluster, miR-21, and miR-217 have been reported to function as oncogenes and miR-181a, miR-34a, miR146a, Cluster miR-15a/16-1, and miR-28 as tumor suppressor genes in B-cell lymphomas [73]. A univariate survival analysis performed in 64 diffuse large B-cell lymphoma patients showed an association of miR-199b expression with a better prognosis and with the germinal center B cell-like (GCB) subtype [74], known to confer a more favorable outcome than the activated B cell-like (ABC) subtype. 

## 3. PODXL in Cancer Cell Survival, Proliferation, and Stemness

The contribution of PODXL to human cancer progression has been demonstrated in a variety of cancer cells by gain- and loss-of-function studies, although the underlying mechanisms remain poorly understood (Table 1).

A role for PODXL in tumor cell proliferation was first evidenced in JHU-0879 glioblastoma multiforme stem-like cell line after silencing of PODXL with specific short hairpin RNAs (shRNAs), which resulted in a significant reduction in cell proliferation and oncosphere formation [45]. In LN-299 and U-118 MG human glioblastoma multiforme cells, ectopic overexpression of PODXL increased soluble/intracellular beta-catenin levels and induced mRNA expression of the beta-catenin signaling target genes *c-MYC* and *c-JUN* and cell proliferation through a mechanism dependent on p38 mitogen- and beta-catenin signaling [75]. Moreover, PODXL increased the level of inhibitory phosphorylation of glycogen synthase kinase-3B (GSK3B) via activation of p38 mitogen-activated protein kinase (MAPK), indicating that PODXL enhances glioblastoma multiforme proliferation by increasing the soluble beta-catenin level/beta-catenin signaling through a mechanism dependent on p38 MAPK/GSK3B pathway [75]. PODXL has also been found to induce proliferation in LN-299 and U-118 MG glioblastoma multiforme cells by inhibiting angiotensin-(1-7)/Mas signaling, known to abrogate growth in many cancer cells [76].

The contribution of PODXL to gastric cancer proliferation has been demonstrated in several studies. In BGC823 and MGC803 gastric cancer cells, PODXL promoted colony formation [77]. Additionally, PODXL in SGC-7901 gastric cancer cells favored proliferation and colony formation, abrogated cell apoptosis, activated phosphatidylinositol 3-kinase (PI3K)/AKT, MAPK/ERK, and NF-kB signaling pathways, and promoted tumorigenesis in a mouse xenograft model through a mechanism dependent on RUN and FYVE domain containing 1 (RUFY1) [52]. The PI3K/AKT pathway is one of the most commonly activated drivers of cancer and promotes tumor initiation and progression [91,92]. PODXL in SGC-7901 and AGS gastric cancer cells also enhanced primary tumor growth in nude mice [51]. In HCT15 colorectal cells, knockdown of PODXL reduced the expression of TAZ protein, its downstream targets survivin, connective tissue growth factor (CTGF), CYR61 and cyclinD1, and stem-cell-related genes, as well as tumorsphere formation, indicating that PODXL plays a crucial role in self-renewal of colon cancer cells [54].

Knockdown of PODXL in HCT116 and LOVO colorectal cancer cell lines suppressed cell proliferation and clonogenic potential, promoted apoptosis, and increased protein levels of caspase-3 and caspase-9, pointing to a role for PODXL in cell survival [78].

In SAS human oral squamous cell carcinoma cell line, silencing of PODXL abrogated cell proliferation and colony formation [46]. This effect was corroborated in vivo by transplanting PODXL-silenced HSC-2 oral squamous cell carcinoma cell line into nude mice, which resulted in both tumor volume and tumor weight reduction compared to that derived from parental HSC-2 cells [79]. 

Contrasting with these data, in the breast cancer cell lines MDA-MB-231, the highly aggressive MDA-MB-231 clone 4175 and NAMEC8R, all expressing high levels of endogenous PODXL, silencing of PODXL exerted no effect on cell proliferation under monolayer culture conditions [80,81,82]. However, the frequency of tumorsphere-forming cells was markedly decreased in PODXL-silenced MDA-MB-231 breast cancer cell line and, conversely, its overexpression in luminal-like MCF-7 breast cancer cell line, a low metastatic cell line expressing low levels of endogenous PODXL, resulted in increased tumorsphere formation [80]. Consistent with these results, silencing of PODXL in MDA-MB-231 cells reduced primary tumor growth in a mouse model xenograft [80,82]. Nevertheless, no effect of PODXL silencing on tumor growth was observed when the xenografted cell lines were MDA-MB-231 clone 4175 cells, NAMEC8R, or the pancreatic cancer cell lines SW1990 and Pa03c [81,83]. Of note, a monoclonal antibody that preferentially bound to PODXL expressed on human tumor cells delayed tumor growth and metastasis to the lung in a mouse model using MDA-MB-231 breast cancer cells [80]. All these data demonstrate the complex and crucial role of PODXL in tumor cell proliferation and tumorsphere formation in vitro as well as in primary tumor growth in vivo.

In Raji Burkitt lymphoma cells, we showed that ectopic overexpression of PODXL enhanced cell proliferation and colony formation [27]. Furthermore, overexpression of PODXL in Raji cells induced cell-to-cell adhesion, resulting in the formation of large cell aggregates, a process that was partially abolished by a specific antibody against integrin subunit beta2 [27]. Engagement of lymphocyte function-associated antigen 1 (LFA-1), an adhesion molecule belonging to the subgroup of beta2-integrins, with its ligand intercellular adhesion molecule 1 (ICAM-1) has been reported to inhibit apoptotic cell death in human DND-39 Burkitt lymphoma cell line [93]. 

## 4. PODXL in Metastasis

Metastasis is a complex and multistep process which involves tumor cell dissociation from the primary tumor, invasion of the surrounding extracellular matrix, intravasation through the endothelium into the bloodstream, and extravasation to secondary sites via attaching to endothelial cells and crossing the blood vessel walls. Finally, malignant cells survive and growth at these metastatic sites [94,95]. In order to invade and disseminate, tumor cells utilize dynamic actin-rich membrane protrusions named invadopodia which contain matrix proteases that degrade the extracellular matrix [94,96]. In contrast to metastasis of solid cancers, which requires the acquisition of a metastatic phenotype, lymphoma dissemination is thought to be driven by physiological mechanisms governing normal lymphocyte trafficking [97,98]. In any case, both metastasis and lymphoma dissemination involve the participation of a variety of adhesion molecules, including integrins and selectins, as well as chemokines [97,98].

Several studies provide evidence of a role for PODXL in cancer metastasis in vitro and in vivo (Table 1). In MCF-7 breast cancer cells, forced expression of PODXL perturbed cell–cell junctions, a process which could facilitate breast carcinoma invasion [22]. Additionally, PODXL has been shown to induce collective tumor migration and invasion, as well as tumor budding of MCF-7 cells both in vitro and in vivo [84]. Furthermore, in MCF-7 breast cancer and P3C prostate cancer cell lines, PODXL enhanced cell migration and invasion, matrix metalloproteinase 1 and 9 expression, and activation of MAPK and PI3K activity through its interaction with ezrin in in vitro assays [85]. In the highly aggressive MDA-MB-231 breast cancer cell line, suppression of PODXL decreased invadopodia formation and activation [82]. On the other hand, PODXL overexpression in MCF-7 breast cancer cell line stimulated invadopodia formation and activation, through the induction of Rac1/Cdc42/cortactin signaling [82]. The migratory and invasive properties promoted by PODXL has also been demonstrated in vitro in colorectal cancer (HCT116, LOVO and HCT15), gastric cancer (SGC-7901, AGS, BGC823, and MGC803), malignant testicular tumor (NT2), oral squamous cell carcinoma (SAS), lung adenocarcinoma (A549), and glioblastoma multiforme (LN-299 and U-118) cell lines [32,46,51,54,75,76,77,78,87]. Besides, silencing of PODXL in both NAMEC8R and the highly metastatic MDA-MB-231 4175 breast cancer cells decreased extravasation in vitro, an effect which was totally reversed by overexpressing wild type PODXL [81]. PODXL silencing also decreased the extravasation of MiaPaca2 and Panc1 pancreatic carcinoma cell lines [81].

A study reported that PODXL interacts with the chemokine receptor CXCR4 and promotes CXCL12-mediated migration of mouse primary hematopoietic cells [99]. CXCL12 is a chemokine produced by stromal cells of lymph nodes, bone marrow, liver, lung, and Peyer´s plaques and involved in hematopoietic cell trafficking by binding to CXCR4 expressed on these cells [100]. The CXCL12/CXCR4 axis has been found to play a major role in tumor progression, metastasis, and survival [100,101]. High levels of CXCR4 expression have been detected in B-NHL with wide dissemination to lymph nodes and associated with poor clinical outcome [102,103,104,105]. Recently, we have demonstrated that overexpression of PODXL in Raji Burkitt lymphoma cells increased migration towards CXCL12 [27]. 

A critical event of metastatic dissemination to distant sites is the adhesion of circulating malignant cells to vascular endothelial cells [95]. Many studies point to a role for E-selectin displayed on vascular endothelial cells in the recruitment of tumor cells to metastatic sites in breast, bladder, gastric, pancreatic, and colorectal carcinoma, as well as hematological malignancies [95,106,107]. Interestingly, PODXL has been implicated in the interaction of tumor cells to E-selectin as well as to L-selectin [34]. Silencing of PODXL with specific shRNAs markedly reduced the binding of SW1990 pancreatic tumor cells to immobilized E- and L-selectin under physiological flow conditions, indicating a functional role for PODXL in this process [34]. 

The contribution of PODXL to tumor metastasis to distal sites has been elucidated in vivo in a few studies (Table 1). Overexpression of PODXL in HMLER cells enhanced cell extravasation in the chick CAM assay, an in vivo model for extravasation [81]. In NAMEC8R, MDA-MB-231, and MDA-MB-231 clone 4175 breast cancer cells, knockdown of PODXL significantly inhibited tumor dissemination to distant organs in murine xenograft models [80,81,82], an effect rescued by re-expressing wild type PODXL [81]. In SGC-7901 and AGS gastric cancer cells, silencing of PODXL impaired liver metastasis in nude mice [51]. Recently, depletion of PODXL has been found to reduce liver metastasis in a hemispleen mouse model using SW1990 and Pa03c pancreatic adenocarcinoma cells [83]. This study showed that the direct interaction of PODXL with the large GTP-ase dynamin-2 regulates cytoskeleton dynamics, promoting migration and metastasis of pancreatic cancer cells [83].

## 5. PODXL in EMT

Accumulating evidence supports a critical role of EMT process in driving tumor metastatic dissemination, drug resistance, and immunosuppression [81,108]. During EMT, epithelial cells lose their apical-basal polarity and cell-to-cell contacts, adopting a mesenchymal morphology and migratory and invasive properties [109]. Therefore, the contribution of PODXL to EMT process has been explored in some studies (Table 1). In A549 lung adenocarcinoma cell line, PODXL expression increased during transforming growth factor-beta (TGF-beta)-induced EMT [86], and PODXL silencing reduced morphological changes and molecular markers associated with EMT [86]. Accordingly, forced expression of PODXL in A549 cells promoted changes characteristic of EMT through a process dependent on the activation of PI3K/AKT signaling pathway [87]. Similarly, PODXL silencing in HCT15 colon cancer cells and in SGC-7901 and AGS gastric cancer cells led to a reduction of EMT-associated markers [51,54]. Moreover, the analysis of mRNA expression levels in patients with colon cancer using GSE17536 datasets revealed a positive correlation of PODXL expression with the mesenchymal markers vimentin, N-cadherin, TWIST2, SLUG, and ZEB1 and a negative correlation with the epithelial marker E-cadherin [54]. In a study performed in HMLER human mammary epithelial cells, activation of EMT program by Dox-inducible expression of the EMT transcription factors Snail or ZEB1 resulted in increased mRNA PODXL levels, indicating that PODXL is induced during EMT process [81]. Further, compared to HMLER cells, total and cell surface PODXL protein expression was shown to be upregulated in NAMEC8R cells, which are mammary mesenchymal epithelial cells that naturally arise from HMLER cells [81]. Nevertheless, overexpression of PODXL in HMLER cells did not induce EMT program, indicating that PODXL acts as an effector, but not as an activator, of the EMT program [81]. Interestingly, the authors also showed that PODXL promotes extravasation during EMT by directly engaging the cytoskeletal linker protein ezrin to establish the dorsal cortical polarity necessary for efficient transendothelial migration [81].

Although the role of EMT-related processes in non-epithelial cancers, including lymphoma and leukemia, remains largely unexplored, various EMT transcription factors have emerged as effectors of malignant progression in these diseases [110,111]. In patients with diffuse large B-cell lymphoma, ZEB1 expression has been associated with adverse clinical presentation and poor outcome [112]. More recently, ZEB1 has been shown to be upregulated in diffuse large B-cell lymphoma tissues and cell lines and involved in a positive feedback loop that promotes diffuse large B-cell lymphoma progression and immune evasion [113]. In mantle cell lymphoma patients, high expression levels of ZEB1 were correlated with shorter overall survival [114]. Moreover, knockdown of the EMT activator ZEB1 using specific shRNAs in Granta-519 and/or Jeko-1 mantle cell lymphoma cell lines reduced cell viability, proliferation, and drug resistance and greatly diminished tumor growth in mouse xenograft models, indicating the mediation of ZEB1 in mantle cell lymphoma progression [114]. 

## 6. PODXL in Drug Resistance

A few studies have examined the involvement of PODXL in tumor cell resistance to both conventional cytotoxic agents and immunotherapy drugs (Table 1). Knockdown of PODXL in HCT15 colon carcinoma cells markedly increased the sensitivity to 5-fluorouracil, an inhibitor of thymidylate synthase, and to irinotecan **[54]**, a topoisomerase I inhibitor with reported clinical activity against relapsed or refractory B-NHL in combination chemotherapy [115,116]. Furthermore, enforced expression and PODXL silencing studies in MG-63 and U2OS osteosarcoma cell lines showed that PODXL induces cisplatin chemoresistance via PI3K/AKT signaling pathway [88]. Cisplatin is a chemotherapeutic drug effective against many types of cancers, including NHL [117]. In SCC-4 and Tca8113 oral tongue squamous carcinoma cell lines, PODXL conferred resistance to cisplatin by increasing mRNA stability and protein expression levels of B-cell-specific lymphoma Moloney murine leukemia virus integration site 1 homolog (BMI-1) by means of focal adhesion kinase (FAK) [89]. In SW1783 and U-87 astrocytoma cell lines, PODXL increased cell survival against apoptosis induced by temozolomide, a DNA-alkylating agent widely used as standard therapy for glioblastoma multiforme, through the up-regulation of PI3K/AKT signaling pathway [90]. Temozolomide has been proven to display clinical activity in patients with primary central nervous system lymphoma, a rare but aggressive extranodal NHL, most commonly of the B-cell subtype [118]. 

We have recently demonstrated that overexpression of PODXL in Raji Burkitt lymphoma cells decreases dexamethasone- and hydrogen peroxide-induced cell apoptosis [27]. Dexamethasone is a glucocorticoid included in several chemotherapy protocols for hematological malignancies such as B-cell lymphomas and leukemia, but prolonged use can lead to the development of drug resistance [119,120,121]. Glucocorticoids trigger lymphoma cell apoptosis through the generation of hydrogen peroxide [122], a reactive oxygen species that induces apoptosis and senescence [123]. We have also shown that forced expression of PODXL in Raji Burkitt lymphoma cells increased cell survival upon treatment with obinutuzumab [27], a novel type II glycoengineered humanized anti-CD20 monoclonal antibody with superior ability to induce direct, non-complement dependent cell death and enhanced antibody-dependent cellular cytotoxicity (ADCC) compared to rituximab [124,125]. Obinutuzumab induces cell death through a non-apoptotic mechanism mediated by lysosomes and dependent on actin reorganization [126]. 

## 7. PODXL in Cancer Cell Metabolism

Tumor cells upregulate the expression of nutrient transporters and alter their metabolism to increase the synthesis of proteins, lipids, nucleic acids, and bioenergetic molecules to foster their accelerated proliferation, as well as the production of redox molecules, to protect cells from apoptosis, leading to tumor dependency on specific nutrients [127]. Metabolic rearrangements also influence tumor metastasis, drug response and favor the escape from immune surveillance [128,129], representing potential therapeutic targets [130]. Tumor cells mainly utilize glucose as a primary nutrient source [127]. Numerous cancer cells also increase the rate of glutamine uptake and glutaminolysis for the generation of biosynthetic precursors, the activation of signaling pathways and the maintenance of mitochondria integrity [131,132]. In tumor cells deprived of glucose, glutamine serves as an alternative substrate for the generation of energy and biomolecules. Glutamine metabolism has been reported to play a crucial role in cell survival and proliferation under glucose-starved conditions in a MYC-inducible human Burkitt lymphoma cell line (P493) [133]. 

The participation of PODXL in cell metabolism remains unexplored. PODXL has been reported to upregulate and form a complex with the glucose-transporter 3 (GLUT3) in embryonal carcinoma cancer stem cells [134]. On the other hand, glucose has been found to modulate PODXL expression in both normal and malignant cells [27,135,136]. In HGEC human glomerular epithelial cells, the presence of high-glucose levels downregulated PODXL expression [135,136], which reverted to normal values after cell exposure to low-glucose conditions [136]. Accordingly, we observed that Raji Burkitt lymphoma cells cultured in low-glucose conditions (0.5 mM) expressed increased surface levels of PODXL compared to those grown in high-glucose conditions (11 mM) [27].

Recently, we have uncovered a new function for PODXL as a metabolic reprogramming inducer in Raji Burkitt lymphoma cells [27]. As PODXL triggers both MAPK signaling pathway, known to enhance glutamine metabolism and cell growth [137], and PI3K/AKT signaling axis, a pathway that favors cell survival under glucose limiting conditions [138], we examined the role of PODXL in Raji cell glutamine metabolism [27]. We showed that under glucose-deprived conditions, Raji cells overexpressing PODXL exhibited enhanced cell proliferation, whereas in the absence of glutamine their proliferation decreased and total cell death augmented relative to that of Raji control cells [27]. Moreover, PODXL overexpression in Raji cells induced cell death in glutamine-deprived conditions [27]. Accordingly, the presence of Compound 968, a selective inhibitor of glutaminase 1 (GLS1), the first enzyme in glutaminolysis pathway, significantly diminished the proliferation of Raji cell overexpressing PODXL compared to that of Raji control cells [27]. Our results indicate that PODXL promotes glutaminolysis and glutamine dependence but decreases glucose dependence in Raji Burkitt lymphoma cells [27].

Dysregulation of lipid metabolism is considered a hallmark of cancer cells [139,140]. Tumor cells require fatty acids to generate new membranes, signaling molecules, and energy and store the excess as intracellular lipid droplets [139,141]. Increased expression of fatty acid synthase (FASN), the terminal and crucial enzyme in de novo lipogenesis, has been linked to tumor metastasis, chemoresistance, and reduced patient survival in many cancers [142,143]. Lipid metabolism, as well as enzymes involved in lipogenesis, including FASN, has been reported to be dysregulated in Burkitt lymphoma, resulting in the accumulation of multiple lipid vacuoles in the cytoplasm, a morphological characteristic of Burkitt lymphoma cells [144]. We have recently reported that forced expression of PODXL in Raji Burkitt lymphoma cells enhanced the formation of cytosolic lipid droplets [27]. Moreover, the addition of the FASN inhibitor cerulenin to the culture medium led to a reduced proliferation of Raji cells overexpressing PODXL compared to that of control cells, indicating that PODXL shifts the metabolism toward de novo fatty acid synthesis, thereby increasing the dependency of Raji cell proliferation on this pathway [27]. 

Growing evidence indicates that tumor cells divert glycolytic intermediaries into the pentose phosphate pathway (PPP) to generate both pentose phosphates, necessary for the synthesis of nucleotides that support high cell proliferation, and NADPH [145]. This metabolic pathway plays a pivotal role in tumor cell survival, proliferation, and chemoresistance and has been associated with tumor aggressiveness [145]. Accordingly, dysregulation of PPP enzymes such as glucose 6-phosphate dehydrogenase (G6PD), the first and rate-limiting enzyme of this pathway, has been reported to promote tumorigenesis [145]. Several cancer cells, including NHL, exhibit increased expression of G6PD as well as PPP flux, which is correlated with poor prognosis [146,147,148]. We have recently shown that in the presence of 6-aminonicotinamide, a competitive inhibitor of G6PD and PPP, Raji Burkitt lymphoma cells overexpressing PODXL proliferated to a lesser extent than control cells, indicating that PODXL induces PPP flux, becoming dependent on this pathway [27]. 

## 8. Conclusions and Future Perspectives

**Figure 1:**
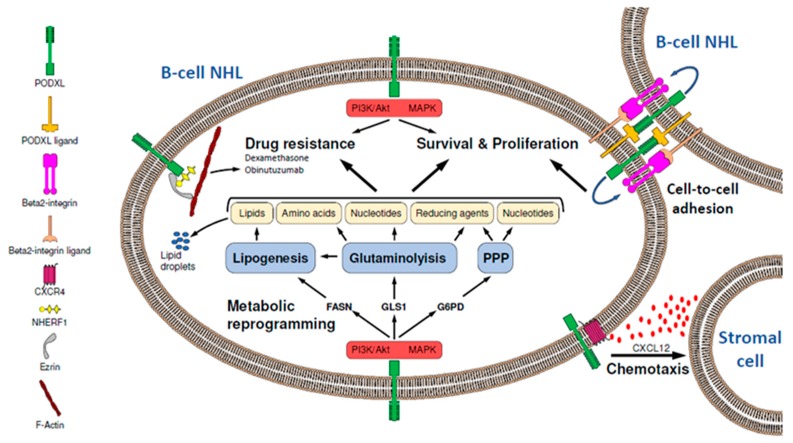
Schematic representation of the potential role of podocalyxin (PODXL) in mature B-cell non-Hodgkin lymphoma (B-NHL) progression. PODXL induces cell survival, proliferation, and drug resistance in mature B- NHL cells, likely through the activation of activated phosphatidylinositol 3-kinase (PI3K)/AKT and mitogen-activated protein kinase (MAPK) signaling pathways. Additionally, PODXL promotes glutaminolysis, lipogenesis, and pentose phosphate pathway via activation of glutaminase 1 (GLS1), fatty acid synthase (FASN), and glucose 6-phosphate dehydrogenase (G6PD), respectively, which would generate biomolecules and reducing agents necessary for tumor cell survival, proliferation, and drug resistance. This metabolic reprogramming may be mediated by PI3K/AKT and MAPK signaling pathways. The PODXL-induced B-NHL-resistance to obinutuzumab could be due to the capacity of PODXL to reorganize the actin cytoskeleton. In addition to this, PODXL favors cell-to-cell adhesion in B-NHL by a beta2-integrin-mediated process, which would lead to cell survival. PODXL also enhances chemotaxis of B-NHL cells towards C-X-C motif chemokine ligand 12 (CXCL12), which is released by stromal cells of distant sites and binds to C-X-C motif chemokine receptor 4 (CXCR4), a chemokine receptor that interacts with PODXL. NHERF1: Na^+^/H^+^-exchanger regulatory factor; PPP: pentose phosphate pathway.

PODXL is overexpressed in various types of cancer and associated with tumor aggressiveness and poor prognosis. Several studies have reported a role for PODXL in regulating critical biological processes that promote tumor progression, including cell proliferation, survival, stemness, EMT, and metastasis, as well as resistance to drugs. In Burkitt lymphoma cells, PODXL induces cell proliferation, survival, clonogenicity, chemotaxis, and resistance to dexamethasone and obinutuzumab and reprograms tumor cell metabolism to maintain its high proliferative activity, yet the underlying molecular mechanisms remain unexplored (Figure 1). 

Our knowledge on the role of PODXL in mature B-NHL progression is based on experiments conducted on a single Burkitt lymphoma cell line. Hence, it remains to be proven whether these findings also apply to other mature B-NHL subtypes. On the other hand, PODXL expression has been determined in malignant cells from a reduced cohort of patients with mature B-NHL. The analysis of a broad array of mature B-NHL samples would allow to establish whether stratification of patients according to PODXL expression predicts patients’ survival and defines a novel subgroup of patients with unfavorable prognosis. PODXL might predict response to therapy, central nervous system relapse, or transformation of indolent follicular lymphoma to aggressive diffuse large B-cell lymphoma. Moreover, PODXL might constitute a potential therapeutic target for the treatment of B-NHL expressing this molecule. In this regard, the expression of tumor-specific PODXL glycoforms would allow the development of blocking antibodies with reduced undesirable side effects. Further studies are still required to decipher the biological function of PODXL in different subtypes of mature B-NHL and the molecular mechanism governing PODXL-induced mature B-NHL progression in order to determine the potential of PODXL as a therapeutic target.

## Figures and Tables

**Table 1:** Role of podocalyxin (PODXL) in human cancer progression.

**Survival-Proliferation-Stemness**				
**Tumor Cell Type**	**Cell Line Model**	**Method**	**Biological Effect and Mechanism**	**Ref.**
Glioblastoma multiforme	JHU-0879	Silencing of PODXL	Decreased proliferation and tumorsphere formation	[45]
	LN-299; U-118 MG	Ectopic overexpression of PODXL	Increased proliferation and *c-MYC* and *c-JUN* mRNA levels (by increasing beta-catenin signaling through the p38 MAPK/GSK3B pathway)	[75]
		Silencing of PODXL	Decreased proliferation	
	LN-299; U-118 MG	Ectopic overexpression of PODXL	Increased proliferation (by inhibiting Ang-(1-7)/Mas signaling through a PI3K dependent mechanism)	[76]
		Silencing of PODXL	Decreased proliferation	
Gastric cancer	BGC823	Ectopic overexpression of PODXL	Increased colony formation	[77]
	MGC803	Silencing of PODXL	Decreased colony formation	
	SGC-7901	Ectopic overexpression of PODXL	Increased cell proliferation, colony formation and activation of PI3K/AKT, NF-kb, MAPK (through interaction with RUFY1) Decreased apoptosis	[52]
		Silencing of PODXL	Decreased cell proliferation, colony formation and activation of PI3K/AKT, NF-kb, MAPK Increased apoptosis	
		Ectopic overexpression of PODXL/Mouse xenograft	Increased tumor growth in vivo (through interaction with RUFY1)	
		Silencing of PODXL/Mouse xenograft	Decreased tumorigenesis in vivo	
	SGC-7901; AGS	Silencing of PODXL/ Mouse xenograft	Decreased tumor growth in vivo	[51]
Colon cancer	HCT15	Silencing of PODXL	Decreased tumorsphere formation Decreased TAZ, survivin, CTGF, cyclinD1, and stem-cell-related gene expression	[54]
	HCT116; LOVO	Silencing of PODXL	Decreased proliferation and clonogenic potential Increased apoptosis and caspase-3 and caspase-9 expression	[78]
Oral squamous cell carcinoma	SAS	Silencing of PODXL	Decreased proliferation and colony formation	[46]
	HSC-2	Silencing of PODXL	Decreased proliferation	[79]
		Silencing of PODXL/mouse xenograft	Decreased tumor growth in vivo	
Breast cancer	MCF-7	Ectopic overexpression of PODXL	Increased formation of tumorspheres	[80]
	MDA-MB-231	Silencing of PODXL	No effect on cell proliferation under adherent culture conditions Decreased formation of tumorspheres	
		Silencing of PODXL/Mouse xenograft	Decreased primary tumor growth, invasion and distant metastasis, in vivo	
	MDA-MB-231 (clone 4175); NAMEC8R	Silencing of PODXL	No effect on cell proliferation under culture conditions	[81]
		Silencing of PODXL/Mouse xenograft	No effect on primary tumor growth in vivo	
	MCF-7	Ectopic overexpression of PODXL	Increased formation of tumorspheres	
	MDA-MB-231	Silencing of PODXL	No effect on cell proliferation under culture conditions	[82]
		Silencing of PODXL/Mouse xenograft	Decreased primary tumor growth	
Pancreatic cancer	SW1990; Pa03c	Silencing of PODXL/Mouse xenograft	No effect on primary tumor growth in vivo	[83]
Burkitt lymphoma	Raji	Ectopic overexpression of PODXL	Increased proliferation and colony formation	[27]
**Metastasis**				
**Tumor Cell Type**	**Cell Line Model**	**Method**	**Biological Effect and Mechanism**	**Ref.**
Glioblastoma multiforme	LN-299; U-118 MG	Ectopic overexpression of PODXL	Increased invasion and MMP-9 expression and activation (by inhibiting Ang-(1-7)/Mas signaling through a PI3K dependent mechanism)	[76]
		Silencing of PODXL	Decreased invasion	
	LN-299; U-118 MG	Ectopic overexpression of PODXL	Increased invasion (by increasing beta-catenin signaling through p38 MAPK pathway)	[75]
		Silencing of PODXL	Decreased invasion	
Gastric cancer	SGC-7901; AGS	Silencing of PODXL	Decreased migration and invasion Decreased expression of MMP-2	[51]
		Silencing of PODXL/Mouse xenograft	Decreased liver metastasis, in vivo	
	SGC-7901; AGS	Ectopic overexpression of PODXL	Increased migration and invasion	[52]
		Silencing of PODXL	Decreased migration and invasion	
	BGC823	Ectopic overexpression of PODXL	Increased migration and invasion	[77]
	MGC803	Silencing of PODXL	Decreased migration and invasion	
Colon cancer	HCT116; LOVO	Silencing of PODXL	Decreased migration and invasion	[78]
	HCT15	Silencing of PODXL	Decreased migration and invasion	[54]
Oral squamous cell carcinoma	SAS	Silencing of PODXL	Decreased migration and invasion Inhibition of FAK activation and filopodia and invadopodia formation	[46]
Breast cancer	MCF-7	Ectopic overexpression of PODXL	Perturbation of cell-cell junctions	[22]
	MCF-7	Ectopic overexpression of PODXL	Increased collective migration in 2-D culture (dependent on ezrin) Increased collective budding and invasion in 3-D culture (dependent on actomyosin)	[84]
		Ectopic overexpression of PODXL/Mouse xenograft	Increased collective invasion and tumor budding, in vivo	
	MCF-7	Ectopic overexpression of PODXL	Increased migration and invasion, matrix metalloproteinases 1 and 9 expression, MAPK and PI3K activity (by interacting with ezrin)	[85]
	MCF-7	Ectopic overexpression of PODXL	Increased invadopodia formation (through Rac1/Cdc42/cortactin signaling)	[82]
	MDA-MB-231	Silencing of PODXL	Decreased invadopodia formation	
		Silencing of PODXL/Mouse xenograft	Decreased distant metastasis, in vivo	
	MDA-MB-231	Silencing of PODXL/Mouse xenograft	Decreased invasion and distant metastasis, in vivo	[80]
	MDA-MB-231 (clone 4175); NAMEC8R	Silencing of PODXL	Decreased in vitro extravasation Decreased migration. No effect on invasion	[81]
		Silencing of PODXL/mouse xenograft	Decreased lung metastasis, in vivo	
	HMLER	Ectopic overexpression of PODXL	Increased in vitro extravasation	
		Ectopic overexpression of PODXL/chick CAM assay	Increased in vivo extravasation	
Prostate cancer	P3C	Ectopic overexpression of PODXL	Increased migration and invasion, matrix MMP-1 and MMP-9 expression, MAPK and PI3K activity (by interacting with ezrin)	[85]
Pancreatic adenocarcinoma	MiaPaca2; Panc1	Silencing of PODXL	Decreased in vitro extravasation	[81]
	SW1990; Pa03c	Silencing of PODXL/Mouse xenograft	Decreased lung metastasis, in vivo	[83]
Lung adenocarcinoma	A549	Silencing of PODXL	Decreased migration	[86]
	A549	Ectopic overexpression of PODXL	Increased migration and invasion (through PI3K/AKT pathway)	[87]
		Silencing of PODXL	Decreased migration and invasion	
Testicular cancer	NT-2	Silencing of PODXL	Decreased invasion	[32]
Burkitt lymphoma	Raji	Ectopic overexpression of PODXL	Increased migration towards CXCL12	[27]
**EMT Process**				
**Tumor Cell Type**	**Cell Line Model**	**Method**	**Biological Effect and Mechanism**	**Ref.**
Gastric cancer	SGC-7901; AGS	Silencing of PODXL	Decreased EMT-associated markers	[51]
Colon cancer	HCT15	Silencing of PODXL	Decreased EMT-associated markers	[54]
Breast cancer	MDA-MB-231 (clone 4175); NAMEC8R	Ectopic overexpression of PODXL	No effect on EMT	[81]
		Silencing of PODXL	No effect on EMT	
	HMLER	Ectopic overexpression of PODXL	No induction of EMT	
Lung adenocarcinoma	A549	Ectopic overexpression of PODXL	Increased EMT morphological changes and markers (through PI3K/AKT pathway)	[87]
		Silencing of PODXL	Decreased EMT morphological changes and markers	
	A549	Silencing of PODXL	Decreased EMT morphological changes and markers	[86]
**Resistance to Drugs**				
**Tumor Cell Type**	**Cell Line Model**	**Method**	**Biological Effect and Mechanism**	**Ref.**
Colon cancer	HCT15	Silencing of PODXL	Increased sensitivity to 5-fluorouracil and to irinotecan	[54]
Osteosarcoma	MG-63; U2OS	Ectopic overexpression of PODXL	Increased resistance to cisplatin (by PI3K/AKT pathway)	[88]
		Silencing of PODXL	Increased sensitivity to cisplatin	
Oral tongue squamous carcinoma	SCC-4; Tca8113	Ectopic overexpression of PODXL	Increased resistance to cisplatin (by increasing BMI-1 and FAK)	[89]
		Silencing of PODXL	Increased sensitivity to cisplatin	
Astrocytoma	SW1783	Ectopic overexpression of PODXL	Increased resistance to temozolomide (by PI3K/AKT pathway)	[90]
	U-87	Silencing of PODXL	Increased sensitivity to temozolomide	
Burkitt lymphoma	Raji	Ectopic overexpression of PODXL	Increased resistance to dexamethasone and obinutuzumab	[27]
**Cancer Cell Metabolism**				
**Tumor Cell Type**	**Cell Line Model**	**Method**	**Biological Effect and Mechanism**	**Ref.**
Burkitt lymphoma	Raji	Ectopic overexpression of PODXL	Increased lipogenesis, PPP, glutaminolysis, and glutamine dependence; decreased glucose dependence	[27]

Ang-(1-7)/Mas: angiotensin-(1-7)/Mas; BMI-1: B-cell-specific lymphoma Moloney murine leukemia virus integration site 1 homolog; CTGF: connective tissue growth factor; CXCL12: C-X-C motif chemokine ligand 12; 2-D: two-dimensional; 3-D: three-dimensional; EMT: epithelial-mesenchymal transition; FAK: focal adhesion kinase; GSK3B: glycogen synthase kinase-3B; MAPK: mitogen-activated protein kinase; PI3K: phosphatidylinositol 3-kinase; PODXL: podocalyxin; PPP: pentose phosphate pathway; RUFY1: RUN and FYVE domain containing 1.

## Abbreviations

**Abb.**	**Full Name**
ADCC	Antibody-dependent cellular cytotoxicity
BMI-1	B-cell-specific Moloney murine leukemia virus integration site 1 homolog
B-NHL	B-cell non-Hodgkin lymphoma
CXCR4	C-X-C motif chemokine receptor 4
CXCL12	C-X-C motif chemokine ligand 12
EMT	Epithelial-mesenchymal transition
FASN	Fatty acid synthase
G6PD	Glucose-6-phosphate dehydrogenase
GLUT3	Glucose transporter 3
GSK3B	Glycogen synthase kinase-3B
KLF4	Kruppel-like factor 4
MAPK	Mitogen-activated protein kinase
NHL	Non-Hodgkin lymphoma
PI3K	Phosphatidylinositol 3-kinase
PINCH1	Particularly interesting new cysteine-histidine rich protein 1
PODXL	Podocalyxin
PPP	Pentose phosphate pathway
shRNAs	Short hairpin RNAs
SP1	Specific protein 1
TGF-beta	Transforming growth factor-beta
WT1	Wilms´ tumor antigen 1
